# A computational peptide model induces cancer cells’ apoptosis by docking Kringle 5 to GRP78

**DOI:** 10.1186/s12860-023-00484-3

**Published:** 2023-08-08

**Authors:** Ibrahim Khater, Aaya Nassar

**Affiliations:** 1 Biophysics Department, Faculty of Science, Cairo University, Giza, Egypt; 2 Department of Clinical Research and Leadership, School of Medicine and Health Sciences, George Washington University, Washington DC, USA

**Keywords:** Cancer, GRP78, Kringle 5, Apoptosis, Molecular docking

## Abstract

**Background:**

Cells can die through a process called apoptosis in both pathological and healthy conditions. Cancer development and progression may result from abnormal apoptosis. The 78-kDa glucose-regulated protein (GRP78) is increased on the surface of cancer cells. Kringle 5, a cell apoptosis agent, is bound to GRP78 to induce cancer cell apoptosis. Kringle 5 was docked to GRP78 using ClusPro 2.0. The interaction between Kringle 5 and GRP78 was investigated.

**Results:**

The interacting amino acids were found to be localized in three areas of Kringle 5. The proposed peptide is made up of secondary structure amino acids that contain Kringle 5 interaction residues. The 3D structure of the peptide model amino acids was created using the PEP-FOLD3 web tool.

**Conclusions:**

The proposed peptide completely binds to the GRP78 binding site on the Kringle 5, signaling that it might be effective in the apoptosis of cancer cells.

## Introduction

Cancer is a major global public health concern with its rising incidence over the past few decades, and it has significant economic and social impacts [1, 2]. This rise in cancer incidence poses a significant challenge to the healthcare industry, as effective therapeutic treatment options are often limited, and the development of new treatments is complex and time-consuming. One of the Sustainable Development Goals of the United Nations for 2030 [3] is to reduce the burden of cancer through prevention and new treatment methods. During the COVID-19 outbreak, which has caused delays and interruptions in cancer screenings, diagnoses, and treatments around the world, it has been especially important to coordinate efforts to reduce cancer burden.

Utilizing computational analysis methods, specifically molecular dynamic simulation, is one method for addressing the problem of therapeutic cancer treatment [4–7]. The computational approach enables researchers to investigate the atomic-level behavior of molecules and gain insight into the molecular mechanisms that drive cancer progression and treatment response. By using molecular dynamic simulation to study the interactions between cancer cells and potential therapeutic agents, researchers can identify new targets for drug development and optimize existing treatment strategies [8–10].

Anticancer therapies have been focused on their ability to destroy cancer cells while preserving healthy cells. Targets for anticancer treatments are molecules that are selective for cancer cells. Peptides that target cancer cells could be used in the development of anticancer drug delivery agents for localizing cancer sites [7, 11]. On the surface of cancer cells, the glucose-regulated protein 78 (GRP78) exhibits enhanced expression [12, 13]. GRP78, located on the endoplasmic reticulum, has been shown to be up-regulated in a number of cancerous tumors [9, 14, 15]. GRP78 is commonly known as binding immunoglobulin protein (BiP) or the heat shock protein 70 kDa family A member 5 (HSPA5) and is encoded by the HSPA5 gene in humans [16]. HSPA5 belongs to the HSP70 chaperon family and is found mostly in the endoplasmic reticulum (ER) lumen [17]. GRP78 usually binds to the ER stress sensors ATF-6, PERK, and IRE1 and deactivates them. In addition, GRP78 is shifted away from the stress sensors and toward the plasma membrane as a result of ER stress, which generates an accumulation of unfolded proteins. GRP78 is extensively expressed on the cell surfaces of a number of cancer types due to their fundamentally elevated ER stress levels, whereas it is relatively weakly expressed on normal cells [16, 18, 19].

When natural ligands bind to GRP78 on the cell surface, they can cause a number of signaling responses. GRP78 can then bind to 2-macroglobulin and Cripto on tumor cells, causing cell survival and proliferation, or bind to Kringle 5 and Par-4, causing cell death [14, 19, 20]. Many studies have focused their efforts on finding small compounds that can bind to cell surface GRP78 and activate apoptotic pathways as an effective anticancer treatment because cell surface GRP78 agonists can trigger an apoptotic response [8, 10]. Arap et al. (2004) investigated the use of the stress response chaperone GRP78 as a target for tumor-specific ligands [19]. The authors demonstrated that GRP78 is selectively expressed on the surface of tumor cells and that this expression is increased under conditions of cell stress. They then show that phage display technology can be used to identify ligands that bind specifically to cell surface GRP78 and that these ligands can be used to selectively target tumor cells in vitro and in vivo. Two potential peptides with anticipated GRP78 binding motifs were designed, evaluated, and tested on mice model [19]. The two animal models were used to test the peptides: nude mice with DU145 prostate subcutaneous tumor xenografts and immunocompetent Balb/c mice with EF43-fgf4 tumors. In both animal models, the peptides were able to specifically bind to GRP78, internalize cells, and decrease tumor growth. A similar study disclosed that peptides targeting GRP78 reduce cancer cell growth without harming other organ cells [21]. While anti-cancer delivery agents targeting GRP78 on the cell surface is consistent with previous findings, more studies are needed.

The current study employs computational docking screening to investigate potential Kringle 5 peptides that bind to GRP78 and cause cancer cell apoptosis. Docking of Kringle 5 to GRP78 was performed in silico, and the interacting peptides were studied.

## Materials and methods

### Structural analysis

The structure of Kringle 5 was obtained from the data bank of protein (PDB ID: 5HPG). The protein structures were downloaded from (https://www.rcsb.org/structure/5HPG).

### Docking Kringle 5 to GRP78

The webserver ClusPro 2.0 for protein docking interactions was used to dock Kringle 5 (5HPG: A) to GRP78. ClusPro 2.0 is a powerful tool for predicting the three-dimensional structure of protein–protein complexes through molecular docking simulations. It uses a rigid-body docking approach, in which the two protein structures are kept rigid while their orientations are adjusted to find the optimal binding configuration. The tool, available at (https://cluspro.org), is commonly used for docking analysis of proteins [22–24]. The end-point free energy was used to calculate the binding energies of the complexes. The complexes formed from the docking were sorted in terms of their energy of binding, and calculated using the MM/GBSA free energy analysis of the HawkDock program [25]. The MM/GBSA method combines molecular mechanics (MM) and continuum electrostatics (GBSA) calculations to estimate the free energy of binding based on the molecular interactions between the two proteins. The MM/GBSA free energy analysis involves calculating the binding free energy of the predicted protein–protein complexes by considering the van der Waals, electrostatic, and solvation interactions between the two proteins. This method allows researchers to identify the most stable and physiologically relevant complex structures, and rank the predicted complexes based on their binding affinity. The complex formed with the highest binding energy was then chosen, and the interacting residues of Kringle 5 and GRP78 were identified.

### Peptide model design

We used ESPript (https://espript.ibcp.fr/ESPript/ESPript), a web-based program for extracting and producing an automated analysis of protein structures, with high-quality representations of multiple sequence alignments of proteins. The program reveals sequence similarities, generates sequence logos, secondary structure, and residue conservation plots, and offers ways to enhance and improve the way they are represented [26]. ESPript version 3 was used to align the amino acid sequences of Kringle 5 with the secondary structure of Kringle 5. The sequence of the secondary structure that contains the most relevant and nearable Kringle 5 interacting residues with GRP78 was obtained.

### Physicochemical properties and solubility prediction

The online program ProtParam (http://web.expasy.org/protparam) was employed to calculate the candidate peptide characterizations and hydropathicity index, including aliphatic index (pI), instability index, extinction coefficient, and molecular weight. ProtParam calculates several important protein properties, including the molecular weight, theoretical pI, amino acid composition, extinction coefficient, and instability index. It also provides information on the number of positively charged, negatively charged, and polar residues in the protein sequence, as well as the percentage of hydrophobic and hydrophilic residues [27]. To analyze and compare the solubility of peptides in water, we used the online tool Pepcalc (http://pepcalc.com) [28]. Pepcalc calculates the molecular weight, net charge, isoelectric point, amino acid composition, extinction coefficient, and hydrophobicity index. It also predicts the stability of peptides under different pH and temperature conditions, as well as the solubility and aggregation propensity of the peptide.

### Secondary structure prediction

The PSIPRED prediction method (http://bioinf.cs.ucl.ac.uk/psipred) was used to determine the peptide’s secondary structure [29]. PSIPRED works by analyzing the sequence profile of a protein, which is generated by searching a sequence database for proteins that are similar to the target protein. The program then uses a neural network algorithm to predict the probability of each residue in the protein sequence being in an alpha-helix, beta-strand, or coil, with reported accuracies of up to 82% for secondary structure prediction. The stages of the prediction method are generating a sequence profile, predicting the initial secondary structure, and filtering the predicted structure. PSIPRED attempts to normalize the PSIBLAST sequence profile. The initial secondary structure was then predicted using neural networking.

### Tertiary structure prediction and validation

The online computational framework tool PEP-FOLD3 (https://bioserv.rpbs.univ-paris-diderot.fr/services/PEP-FOLD3) was utilized to predict the peptide’s three-dimensional (3D) structure, generating five possible models [30, 31]. The peptides were sorted based on their free energy, which was estimated with Swiss-PdbViewer (https://spdbv.unil.ch). The peptide with the lowest free energy was chosen. Both PROCHECK webserver (https://www.ebi.ac.uk/thornton-srv/software/PROCHECK) [32] and ProSA-web (https://prosa.services.came.sbg.ac.at/prosa.php) [33] were used to verify the best model.

### Docking peptide model to GRP78

The peptide model was docked to GRP78 once more using ClusPro 2.0. The MM/GBSA was used to rank the docking complexes based on their binding energy. The compound with the highest binding energy was selected and searched for interaction residues. The Protein Data Bank in Europe: Protein Interfaces, Surfaces, and Assemblies (PDBePISA), which is a webserver tool available at (https://www.ebi.ac.uk/msd-srv/prot_int/cgi-bin/piserver) was used to investigate the interactions between the proposed peptide and GRP78. PDBePISA works by analyzing the 3D structures of proteins and other molecules in the PDB, and identifying the interfaces and surfaces that are involved in protein–protein, protein–ligand, and protein-DNA interactions. The program then calculates the binding energies and other physical and chemical properties of these interfaces and generates detailed reports and graphical representations of the results.

### Molecular dynamics simulations

The protein topologies and parameter files were created using the web-based graphical user interface CHARMM-GUI [34–36]. The program provides pre-configured simulation protocols for a variety of simulation types, including energy minimization, equilibration, and production runs. The software package GROMACS-2019 [37] was used with the force field CHARMM36 [38], which is a set of mathematical equations and parameters that describe the interactions between atoms in a molecular system, to simulate molecular dynamics. The complexes were then neutralized with the molecular simulation tool TIP3P, which approximates water molecules as three-point charges, with two negative charges at the oxygen atom and one positive charge at the hydrogen atoms, using the solvation box [39], by adding appropriate amounts of K + and Cl ions using the Monte-Carlo ion-placing approach. The system was equilibrated for 125 ps at a stable number of molecules, volume, and temperature (NVT) after the system’s energy was reduced for 5000 steps using the steepest method [40]. Finally, simulations of molecular dynamics were performed.

## Results and discussion

**Fig. 1:**
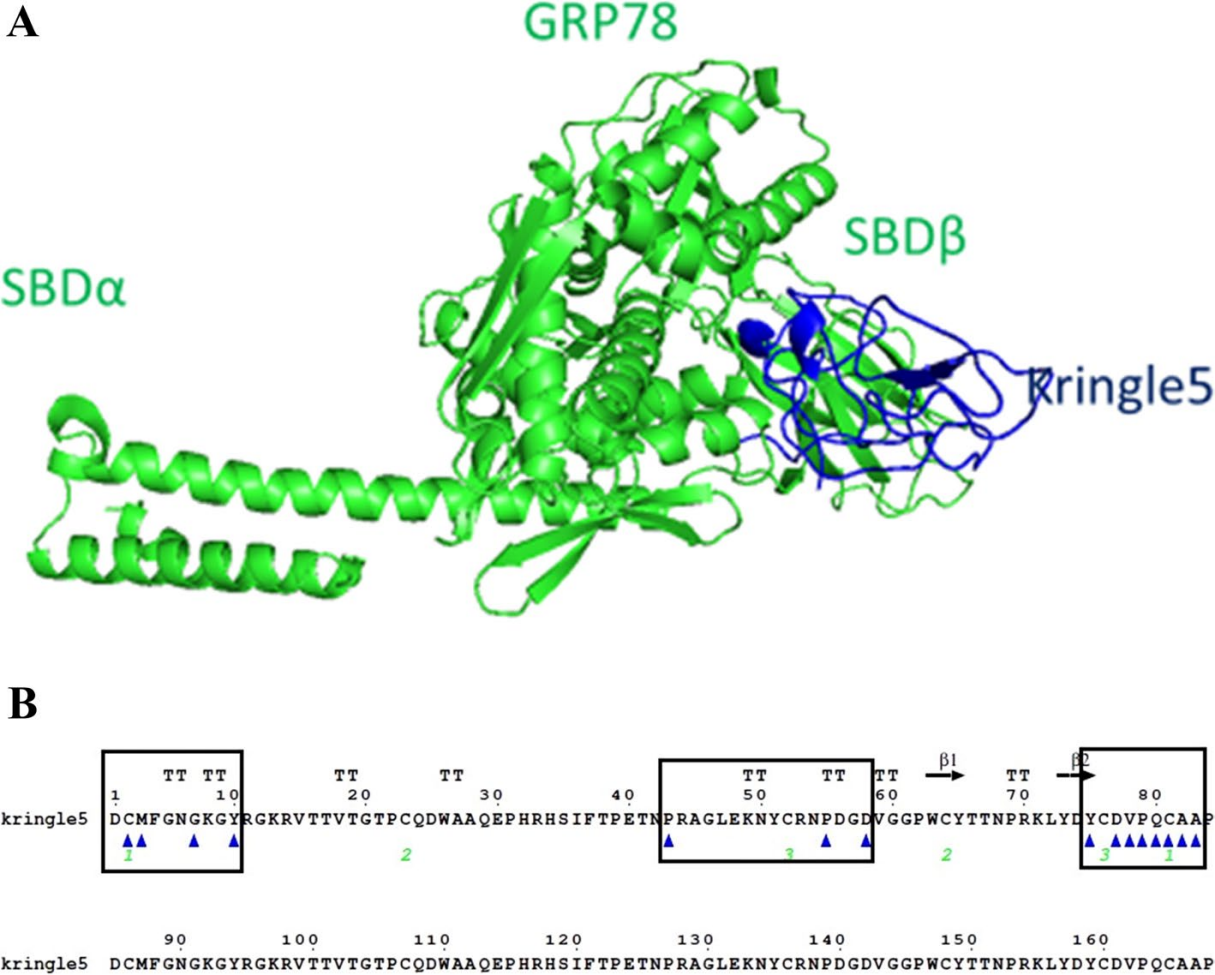
(**A**) Cell apoptosis agent Kringle 5 bound to GRP78. (**B**) The amino acids sequences of GRP78 aligned with its secondary structure using ESPript3 showing interacting amino acids between GRP78 (referred to by blue triangles) and were concentrated in three regions of Kringle 5 (black rectangles surround 35 amino acids)

**Fig. 2:**
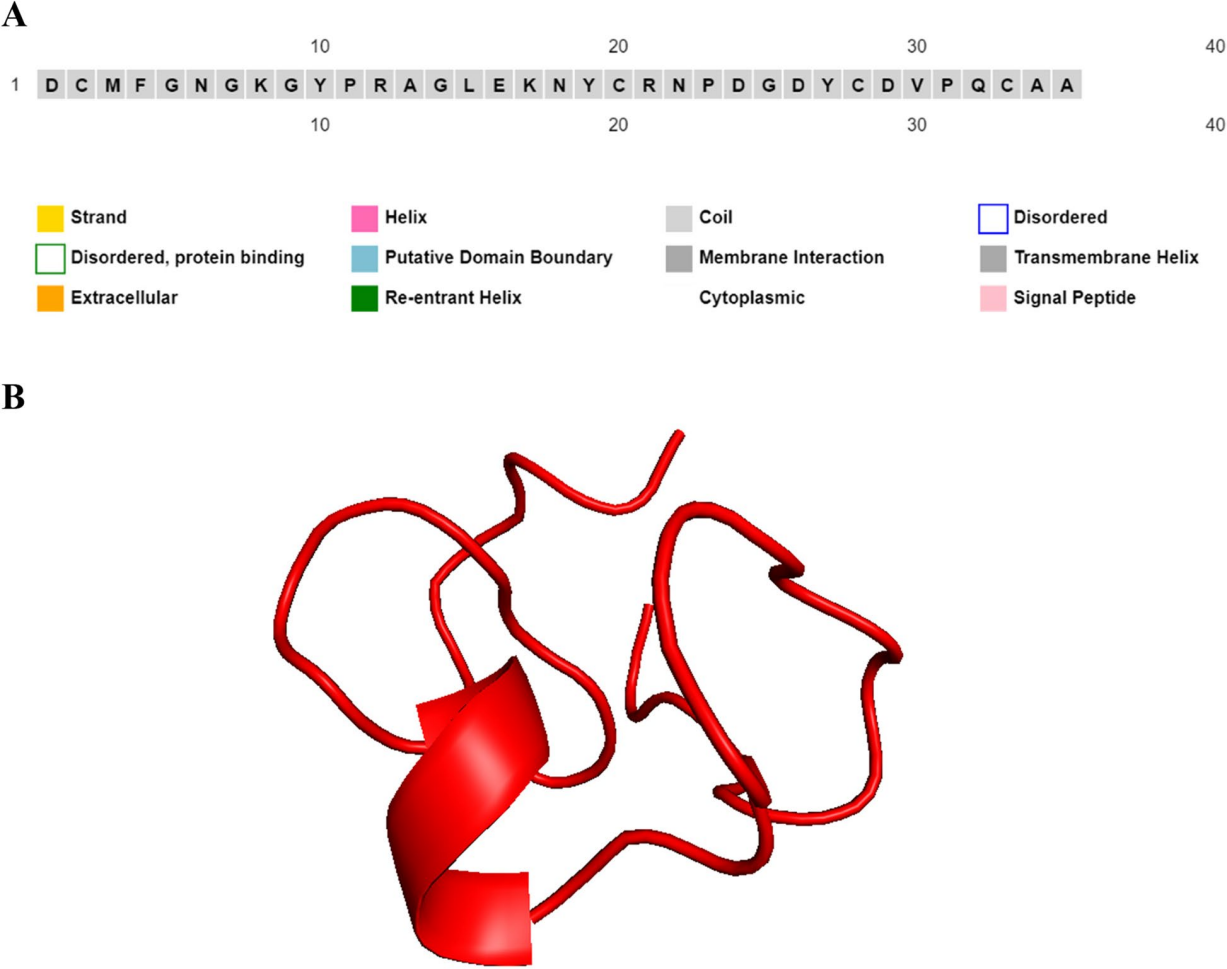
(**A**) PSIPRED predicted secondary structure of final peptide (PEP 35) and (**B**) Predicted 3D structure of final peptide using PEP-FOLD3

ClusPro 2.0 was used to dock Kringle 5, a cell apoptotic agent, to GRP78, as demonstrated in Fig. 1A. The interface residues between Kringle 5 and GRP78 were calculated using the MM/GBSA method and aligned with Kringle 5’s secondary structure using ESPript version 3, as shown in Fig. 1B. The blue triangles indicate interface residues. Interacting amino acids (surrounded by black rectangles) were localized in three regions of Kringle 5. The 35 amino acids contained within the black rectangles were then used to build the peptide model. The peptide has a molecular weight of 3829.24 kDa and a theoretical isoelectric point (pI) of 4.78, which is less than seven, indicating that the protein has a large percentage of negatively charged vs. positively charged residues. It was found that the stability index (Ii) was 21.31, indicating that the solution was stable. The hydrophilic nature of the protein and its ability to interact in aqueous solutions were revealed by the significant average of the hydropathicity index (GRAVY). The aliphatic index (Ai) was 28, suggesting that the peptide could tolerate a broad range of temperatures. Considering that yeast has a half-life of three minutes, mammals’ reticulocytes have a half-life of 1.1 h, and E. coli has a half-life of ten hours. The extinction coefficient (EC) was computed to be 4720 M^−1^ cm^−1^, displaying a great water solubility, and enabling a quantitative assessment of the ligand and protein interactions in solution. Table 1 lists the physicochemical properties of the candidate peptide. Figure 2A shows the proposed peptide’s PSIPRED-predicted secondary structure. Using the PEP-FOLD3 web tool, five models of the peptide’s tertiary structure were created and rated based on free energies. As indicated in Fig. 2B, the best model was chosen and given the name PEP 35.

**Table 1:** The physicochemical properties of the peptide

Sequence	Length	PI	Gravy	MW (Da)	Solubility	Half-life (h)	Ii	Ai	EC (M^-1^ cm^−1^)
DCMFGNGKGYPRAGLEKNYCRNPDGDYCDVPQCAA	35	4.78	-0.883	3829.24	Good water solubility	1.1	21.31	28	4720

**Fig. 3:**
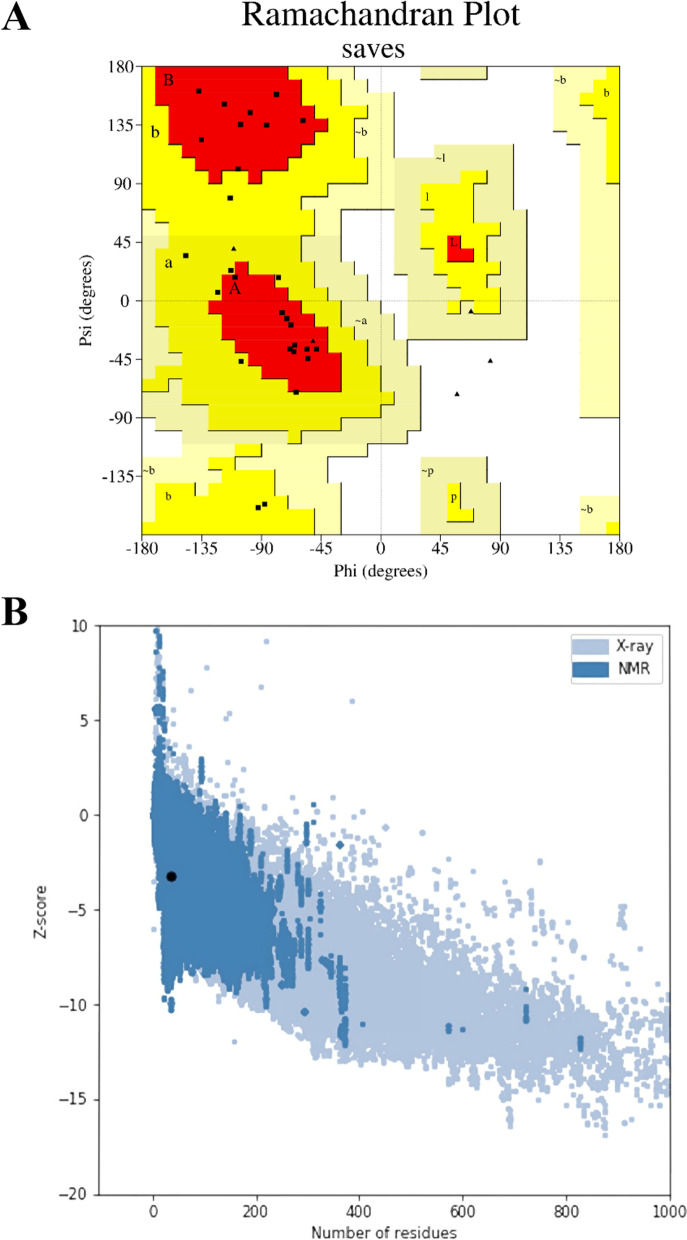
Tertiary structure validation of PEP 35. (**A**) Ramachandran plot of the final peptide modeled, more than 100% of the amino acids are in the allowed regions and (**B**) ProSA-web plot of the peptide, giving a Z-score of -3.25

The Ramachandran plot calculates the energy of each amino acid’s stable conformation psi (ψ) and phi (Ф) twisting or dihedral angles. Ramachandran plot analysis’ tertiary structure validation revealed that the overall percentage of favorable and allowed region residues was 100 percent, as shown in Fig. 3A. After ProSA-web was used to examine the quality and probable flaws in the crude 3D model, the peptide model earned a Z-score of -3.25, as shown in Fig. 3B. The quality of PEP 35 model was confirmed using the Ramachandran plot and the ProSA-web score.

**Fig. 4:**
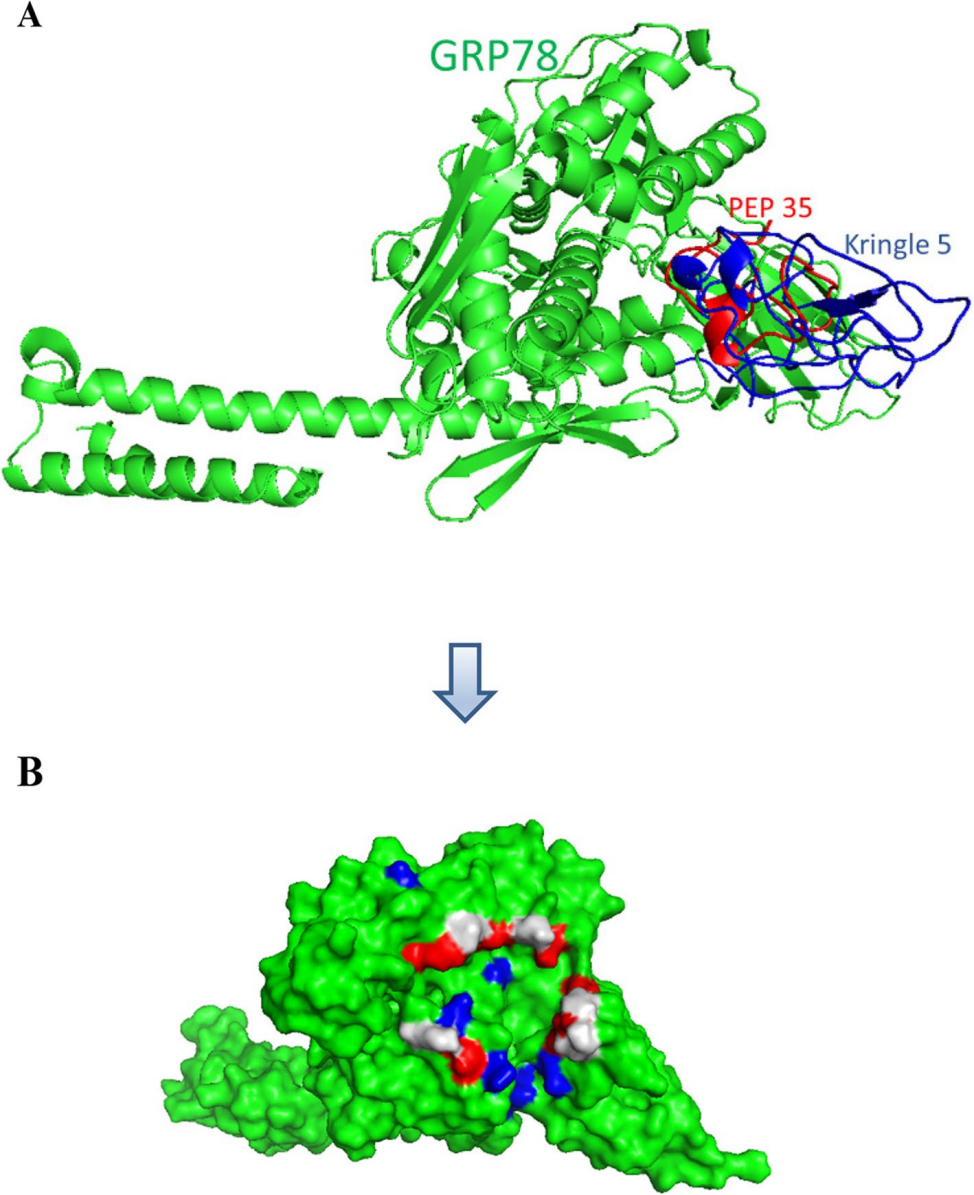
(**A**) PEP 35 (red) bound to GRP78 (green) at the same position of Kringle 5 (blue) (**B**) GRP78 residues interact with PEP35 (in red), Kringle 5 (in blue), and both (in grey)

ClusPro 2.0 was utilized to dock PEP 35 to GRP78. At the same location as Kringle 5, PEP 35 interacts with GRP78. GRP78 residues that interact with both PEP 35 and Kringle 5 are highlighted in grey as demonstrated in Fig. 4A and Fig. 4B, correspondingly. The interactions between the GRP78 and PEP 35 models were studied using PDBePISA, which includes hydrogen bonds and salt bridges, as shown in Table 2. The PEP 35 model developed 19 hydrogen bonds and 6 salt bridges using GRP78. This implies that PEP 35 and GRP78 have a strong interaction.

**Table 2:** The interactions between the GRP78 and PEP 35 analyzed using PDBePISA

PEP 35-GRP78 complex					
Hydrogen bonds			Salt bridges		
GRP78 residue	Peptide residue	Distance (A^o^)	GRP78 residue	Peptide residue	Distance (A^o^)
ASP 105	ASN 6	2.11	ASP 333	ARG 12	3.82
GLN 109	TYR 19	2.24	LYS 340	ASP 1	2.76
LYS 113	ASN 18	1.67	LYS 340	ASP 1	2.52
LYS 113	TYR 19	1.65	LYS 344	ASP 1	2.91
ARG 261	ARG 12	1.81	LYS 344	ASP 1	3.36
ASP 333	ARG 12	2.12	LYS 470	ASP 29	3.82
ASP 333	ARG 12	1.76			
SER 337	ARG 12	2.16			
LYS 340	ASP 1	1.88			
LYS 340	ASP 1	1.76			
LYS 344	ASP 1	2.33			
LYS 344	ASP 1	2.38			
LYS 344	ASP 1	1.91			
LYS 344	MET 3	1.66			
GLU 465	ALA 35	2.02			
GLU 466	CYS 33	2.14			
LYS 470	LYS 8	1.74			
ASP 471	CYS 33	3.80			
ASN 472	LYS 8	1.76			

**Fig. 5:**
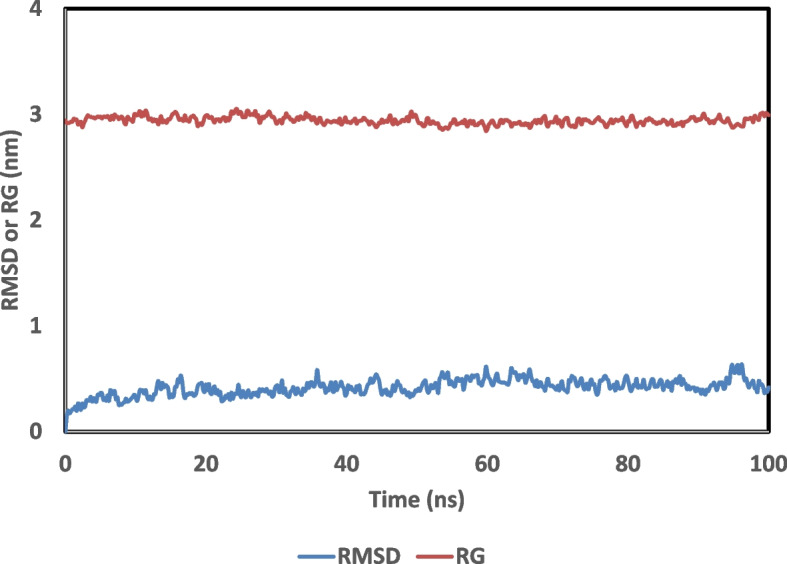
Plot of the Root Mean Square Deviation (RMSD) for the backbone atoms, radius of gyration (RG) of GRP78-PEP 35 complex as a function of time over a 100 ns simulation

The docking mechanism formed binding between the legends and the protein, which could be unstable [41]. The simulations revealed a lot about the molecular interactions that keep the complexes stable. The root mean square difference (RMSD) for the complex backbone GRP78-PEP 35 model was utilized to determine the stability of the complex in comparison to the starting structures. Plotting the gyration radius (RG) as a function of time allowed for the complex’s stability to be ascertained [42]. The RMSD (blue) and RG (red) values of the GRP78-PEP 35 complex are plotted and shown in Fig. 5. The parameters remain steady over the simulation time, indicating that the GRP78-PEP 35 complex is stable. As a result, PEP 35 could be used as an apoptosis agent in cancer cells. The results of the current analysis are in line with the means that GRP78 localizes to the cell surface as previously described by Chen and colleagues [43] and shedding light on the prospect that cancer cell growth would be hindered by new treatments that target GRP78.

## Conclusion

The rising incidence of cancer is a major global public health concern, and it has significant economic and social effects. We demonstrated using molecular docking and molecular dynamics simulations that GRP78 binds to Kringle 5 and triggers the death of tumor cells, indicating that it may be beneficial as a therapeutic agent to site-direct and localize cancer cells. The efficacy of the proposed peptide apoptotic pathway requires additional experimental investigation.

## Data Availability

The Kringle 5 datasets analyzed during the current study are available in the protein data bank (https://www.rcsb.org/structure/5HPG).

## References

[1] Kocarnik JM, Compton K, Dean FE, Fu W, Gaw BL, Harvey JD (2022). Cancer incidence, mortality, years of life lost, years lived with disability, and disability-adjusted life years for 29 cancer groups from 2010 to 2019: a systematic analysis for the global burden of disease study 2019. JAMA Oncol.

[2] Siegel RL, Miller KD, Jemal A (2019). Cancer statistics, 2019. CA Cancer J Clin.

[3] United Nations Development Programme (2023). The SDGs in action. 2030 Sustainable Development Goals.

[4] De B, Bhandari K, Mendonça FJB, Scotti MT, Scotti L (2019). Computational studies in drug design against cancer. Anticancer Agents Med Chem.

[5] Geromichalos GD, Alifieris CE, Geromichalou EG, Trafalis DT (2016). Overview on the current status on virtual high-throughput screening and combinatorial chemistry approaches in multi-target anticancer drug discovery. Part II J BUON.

[6] Geromichalos GD (2007). Importance of molecular computer modeling in anticancer drug development. J BUON.

[7] Hameed R, Khan A, Khan S, Perveen S (2019). Computational Approaches Towards Kinases as Attractive Targets for Anticancer Drug Discovery and Development. Anticancer Agents Med Chem.

[8] Qiao Y, Dsouza C, Matthews AA, Jin Y, He W, Bao J (2020). Discovery of small molecules targeting GRP78 for antiangiogenic and anticancer therapy. Eur J Med Chem.

[9] Abu-Mahfouz A, Ali M, Elfiky A. Anti-breast cancer drugs targeting cell-surface glucose-regulated protein 78: a drug repositioning in silico study. J Biomol Struct Dyn. 2022:1-15. 10.1080/07391102.2022.2125076. 10.1080/07391102.2022.2125076 36129131

[10] Madhavan S, Nagarajan S (2020). GRP78 and next generation cancer hallmarks: an underexplored molecular target in cancer chemoprevention research. Biochimie.

[11] Yoneda Y, Steiniger SCJ, Capková K, Mee JM, Liu Y, Kaufmann GF (2008). A cell-penetrating peptidic GRP78 ligand for tumor cell-specific prodrug therapy. Bioorg Med Chem Lett.

[12] Cicalese S, Okuno K, Elliott KJ, Kawai T, Scalia R, Rizzo V, Eguchi S. 78 kDa Glucose-Regulated Protein Attenuates Protein Aggregation and Monocyte Adhesion Induced by Angiotensin II in Vascular Cells. Int J Mol Sci. 2020;21(14):4980. 10.3390/ijms21144980. 10.3390/ijms21144980 PMC7403992 32679678

[13] Xia S, Duan W, Liu W, Zhang X, Wang Q (2021). GRP78 in lung cancer. J Transl Med.

[14] Araujo N, Hebbar N, Rangnekar VM (2018). GRP78 is a targetable receptor on cancer and stromal cells. EBioMedicine.

[15] Santamaría PG, Mazón MJ, Eraso P, Portillo F (2019). UPR: an upstream signal to EMT induction in cancer. J Clin Med.

[16] Elfiky AA, Ibrahim IM, Ibrahim MN, Elshemey WM (2022). Host-cell recognition of SARS-CoV-2 spike receptor binding domain from different variants. J Infect.

[17] Wang J, Lee J, Liem D, Ping P (2017). HSPA5 Gene encoding Hsp70 chaperone BiP in the endoplasmic reticulum. Gene.

[18] Ni M, Zhang Y, Lee AS (2011). Beyond the endoplasmic reticulum: atypical GRP78 in cell viability, signalling and therapeutic targeting. Biochem J.

[19] Arap MA, Lahdenranta J, Mintz PJ, Hajitou A, Sarkis AS, Arap W (2004). Cell surface expression of the stress response chaperone GRP78 enables tumor targeting by circulating ligands. Cancer Cell.

[20] Burikhanov R, Zhao Y, Goswami A, Qiu S, Schwarze SR, Rangnekar VM (2009). The tumor suppressor par-4 activates an extrinsic pathway for apoptosis. Cell.

[21] Kao C, Chandna R, Ghode A, Dsouza C, Chen M, Larsson A (2018). Proapoptotic Cyclic Peptide BC71 Targets Cell-Surface GRP78 and Functions as an Anticancer Therapeutic in Mice. EBioMedicine.

[22] Vajda S, Yueh C, Beglov D, Bohnuud T, Mottarella SE, Xia B (2017). New additions to the ClusPro server motivated by CAPRI. Proteins Struct Functi Bioinform.

[23] Kozakov D, Hall DR, Xia B, Porter KA, Padhorny D, Yueh C (2017). The ClusPro web server for protein–protein docking. Nat Protoc.

[24] Kozakov D, Beglov D, Bohnuud T, Mottarella SE, Xia B, Hall DR (2013). How good is automated protein docking?. Proteins.

[25] Weng G, Wang E, Wang Z, Liu H, Zhu F, Li D (2019). HawkDock: a web server to predict and analyze the protein-protein complex based on computational docking and MM/GBSA. Nucleic Acids Res.

[26] Robert X, Gouet P (2014). Deciphering key features in protein structures with the new ENDscript server. Nucleic Acids Res.

[27] Wilkins MR, Gasteiger E, Bairoch A, Sanchez JC, Williams KL, Appel RD (1999). Protein identification and analysis tools in the ExPASy server. Methods Mol Biol.

[28] Lear S, Cobb SL (2016). Pep-Calc.com: a set of web utilities for the calculation of peptide and peptoid properties and automatic mass spectral peak assignment. J Comput Aided Mol Des.

[29] McGuffin LJ, Bryson K, Jones DT (2000). The PSIPRED protein structure prediction server. Bioinformatics.

[30] Lamiable A, Thévenet P, Rey J, Vavrusa M, Derreumaux P, Tufféry P (2016). PEP-FOLD3: faster de novo structure prediction for linear peptides in solution and in complex. Nucleic Acids Res.

[31] Thévenet P, Shen Y, Maupetit J, Guyon F, Derreumaux P, Tufféry P (2012). PEP-FOLD: an updated de novo structure prediction server for both linear and disulfide bonded cyclic peptides. Nucleic Acids Res.

[32] Laskowski RA, MacArthur MW, Moss DS, Thornton JM (1993). PROCHECK: a program to check the stereochemical quality of protein structures. J Appl Crystallogr.

[33] Wiederstein M, Sippl MJ (2007). ProSA-web: interactive web service for the recognition of errors in three-dimensional structures of proteins. Nucleic Acids Res.

[34] Jo S, Kim T, Iyer VG, Im W (2008). CHARMM-GUI: a web-based graphical user interface for CHARMM. J Comput Chem.

[35] Kim S, Lee J, Jo S, Brooks CL, Lee HS, Im W (2017). CHARMM-GUI ligand reader and modeler for CHARMM force field generation of small molecules. J Comput Chem.

[36] Jo S, Cheng X, Islam SM, Huang L, Rui H, Zhu A (2014). CHARMM-GUI PDB manipulator for advanced modeling and simulations of proteins containing nonstandard residues. Adv Protein Chem Struct Biol.

[37] Pronk S, Páll S, Schulz R, Larsson P, Bjelkmar P, Apostolov R (2013). GROMACS 4.5: a high-throughput and highly parallel open source molecular simulation toolkit. Bioinformatics.

[38] Huang J, MacKerell AD (2013). CHARMM36 all-atom additive protein force field: validation based on comparison to NMR data. J Comput Chem.

[39] Mark P, Nilsson L (2001). Structure and dynamics of the TIP3P, SPC, and SPC/E water models at 298 K. J Phys Chem A.

[40] Piche SW (1994). Steepest descent algorithms for neural network controllers and filters. IEEE Trans Neural Netw.

[41] Hollingsworth SA, Dror RO (2018). Molecular dynamics simulation for all. Neuron.

[42] Reva BA, Finkelstein AV, Skolnick J (1998). What is the probability of a chance prediction of a protein structure with an rmsd of 6 Å?. Fold Des.

[43] Chen J, Lynn EG, Yousof TR, Sharma H, MacDonald ME, Byun JH (2022). Scratching the Surface— an overview of the roles of cell surface GRP78 in cancer. Biomedicines.

